# Synthesis of Fluorescent Sulfur Quantum Dots for Bioimaging and Biosensing

**DOI:** 10.3389/fbioe.2022.909727

**Published:** 2022-05-16

**Authors:** Hong Ruan, Li Zhou

**Affiliations:** Key Laboratory of New Processing Technology for Nonferrous Metal and Materials (Ministry of Education), Guangxi Key Laboratory of Optical and Electronic Materials and Devices, College of Materials Science and Engineering, Guilin University of Technology, Guilin, China

**Keywords:** fluorescent nanomaterial, metal-free quantum dots, sulfur quantum dots, bioimaging, biosensing

## Abstract

The rapid industrialization has had a serious impact on the environment, leading to an increase in disease and healthcare problems. The development of simple and effective biosensors to achieve specific analyte detection and bioimaging can provide useful information for disease prevention and treatment. Sulfur quantum dots (SQDs), a new class of metal-free fluorescent nanomaterial, are being studied and applied in diagnostic fields such as bioimaging and biosensing due to their advantages of simple synthetic process, unique composition, ultrasmall size, adjustable fluorescence, and low toxicity. This minireview highlights the main synthetic methods to synthesize fluorescent SQDs and their recent progress in cell and tissue imaging, as well as detection of biomolecules, metal ions, and temperature. Finally, the future development and some critical challenges of SQDs as a fluorescent probe in the field of bioimaging and biosensing are also discussed.

## Introduction

In the past few decades, fluorescent quantum dots (QDs) have been the focus of nanomaterials research because of their attractive optical properties and a wide range of potential applications (Fujita et al., 2019; Tavares et al., 2011; Wegner and Hildebrandt, 2015; Zrazhevskiy et al., 2010). Traditional semiconductor QDs are composed of group II-VI or III-V elements and usually contain toxic heavy metal ions, greatly limiting their applications in biological and environmental fields. As such, the development of metal-free fluorescent QDs such as carbon QDs (Chung and Zhang, 2021; Sun et al., 2006; Liao et al., 2016), graphene QDs (Kalita et al., 2016; Zhou et al., 2017), and silicon QDs (Warner et al., 2005) has attracted more and more attention.

In the past 4 years, sulfur QDs (SQDs), a novel class of metal-free fluorescent QDs, in particular, have generated a tremendous interest because of their unique optical, spectroscopic, chemical, and antibacterial properties (Gao et al., 2020; Pal et al., 2020; Shi et al., 2020). Great progress has been made in the synthesis of SQDs since it was first reported in 2018 (Shen et al., 2018) that SQDs could be synthesized directly from cheap and abundant elemental S. Many researchers have devoted themselves to exploiting the application potential of SQDs. Up to now, SQDs have exhibited application potential in numerous fields, such as fluorescence detection (Huang et al., 2021a; Sheng et al., 2021), bioimaging (Arshad et al., 2021; Zhang et al., 2019), light-emitting diodes (Wang H. et al., 2019), fluorescent polymer composites (Song et al., 2020), photocatalysis (Li et al., 2014), and antibacterial materials (Wang et al., 2021). Among them, SQDs have received special attention in bioimaging and biosensing because of their good biocompatibility, low toxicity, and unique surface properties. The utilization of SQDs as a fluorescent probe for bioimaging and biosensing has been widely studied (Liu et al., 2020; Huang et al., 2021b; Han et al., 2021; Lei et al., 2021). In this minireview, we first summarize the main achievement in the synthesis of fluorescent SQDs and then introduce the research progress of SQDs in bioimaging and biosensing. Finally, the challenges and opportunities of SQDs as a fluorescent probe are discussed. We hope that this minireview will enable researchers to better understand SQDs and promote their practical applications in bioimaging and biosensing.

## Synthesis

**FIGURE 1:**
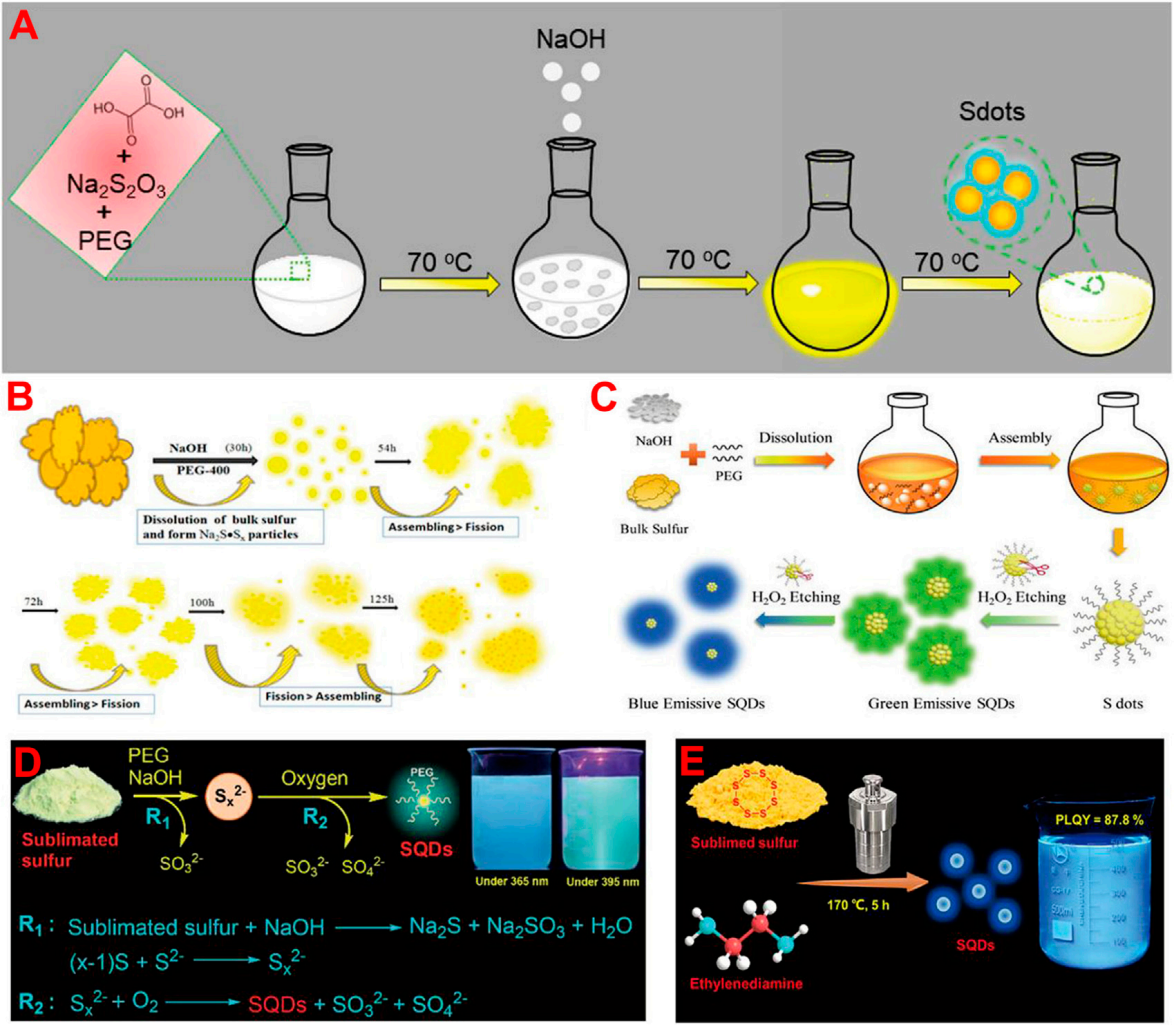
Schematic illustration of the synthesis of fluorescent SQDs by using bottom-up **(A)** and top-down **(B–E)** strategies, respectively. **(A)** Reproduced, with permission, from Arshad and Sk, 2020. Copyright 2020, American Chemical Society. **(B)** Reproduced, with permission, from Shen et al. (2018). Copyright 2018, American Chemical Society. **(C)** Reproduced, with permission, from Wang H. et al. (2019). Copyright 2019, Wiley. **(D)** Reproduced, with permission, from Song et al. (2020). Copyright 2020, Royal Society of Chemistry. **(E)** Reproduced, with permission, from Gao et al. (2022). Copyright 2022, American Chemical Society.

So far, various approaches have been developed for the synthesis of fluorescent SQDs, which can be classified into two categories. The first, called the bottom-up strategy, involves direct oxidation or reduction of sulfur ions in sulfur-containing compounds such as metal sulfides and thiosulfates to zero-valent S atoms. For example, Li and co-workers prepared fluorescent SQDs by oxidizing S^2−^ ions into zero-valent S atoms based on the interfacial reaction between metal sulfide QDs and nitric acid (Li et al., 2014). Sk et al. reported the synthesis of fluorescent SQDs using sodium thiosulfate as the raw material in combination with solution chemistry (Arshad and Sk, 2020) or mechanochemistry (Arshad et al., 2021) and found that this could overcome the shortcoming of requiring a long reaction time when using elemental S directly as the raw material (Figure 1A).

Another synthesis strategy is called the top-down strategy, which is to synthesize fluorescent SQDs directly from elemental S through solution chemistry combined with different reaction conditions such as hydrothermal (Xiao et al., 2019), ultrasonic (Zhang et al., 2019; Zhang et al., 2021), and microwave (Hu et al., 2020) treatment. For example, Shen et al. first reported the preparation of fluorescent SQDs by the reaction of elemental S with NaOH in the presence of a polyethylene glycol (PEG) stabilizer (Shen et al., 2018). They proposed that elemental S first reacted with NaOH to produce water-soluble Na_2_Sx and then formed SQDs through self-assembly and a fission process (Figure 1B). Although the reaction time was very long (125 h) and the fluorescence quantum yield (QY) of the prepared SQDs was low (3.8%), this work opens a door for the direct preparation of fluorescent SQDs from cheap and abundant elemental S (Wang et al., 2020). To date, most reported SQDs were prepared based on the elemental S-NaOH reaction system (Chen et al., 2022; Qiao et al., 2020; Rong et al., 2022; Zhang et al., 2022). Wang H. et al. (2019) found that adding H_2_O_2_ to etch the surface of SQDs could reduce their non-radiative transition and increase the fluorescence QY of SQDs to 23% (Figure 1C). Zhou and co-workers reported that O_2_ could accelerate the reaction rate, which can not only shorten the reaction time to 10 h but also greatly improve the fluorescence QY of SQDs to 21.5% (Song et al., 2020). This is because O_2_ can oxidize Sx^2−^ ions to zero-valent S atoms (Figure 1D). Under the condition of oxygen acceleration, they also synthesized SQDs by using different stabilizers such as carboxymethyl cellulose (CMC) (Duan et al., 2020), polyvinyl alcohol (PVA) (Lei et al., 2021), and hydroxypropyl-β-cyclodextrin (HP-β-CD) (Huang et al., 2021a). It was found that different stabilizers can endow the SQDs with distinct surface properties. Chen et al. prepared SQDs with fluorescence QY of 58.6% by a combination of ultrasonic and microwave treatment with H_2_O_2_ etching (Sheng et al., 2021). In addition to the elemental S-NaOH system, Zhou and co-workers recently reported the solvothermal treatment of elemental S-ethylenediamine solution to prepare highly fluorescent SQDs (Figure 1E). The fluorescence QY of prepared SQDs and the corresponding S-to-SQD conversion efficiency reach as high as 87.8 and 15.9%, respectively, both of which are the highest values to date (Gao et al., 2022). This study provides a new reaction system for large-scale preparation of ultrabright fluorescent SQDs from elemental S.

At present, impressive progress has been made in the direct utilization of elemental S to the scalable synthesis of SQDs with high QY. However, two key problems still need to be solved in the future: 1) the SQDs usually exhibit poor photostability, and it is difficult to withstand long-term light irradiation; 2) the emission wavelength of SQDs is generally located in the short wavelength region and it is easy to be interfered by the self-fluorescence of biological samples. It is well known that the fluorescence lifetime, QY, optical stability, absorption, and emission wavelength of QDs can be effectively adjusted by doping and surface functionalization techniques. Therefore, doping treatment or surface modification of SQDs to tune their properties is an important research direction in the future.

## Cytotoxicity Evaluation and Bioimaging

**FIGURE 2:**
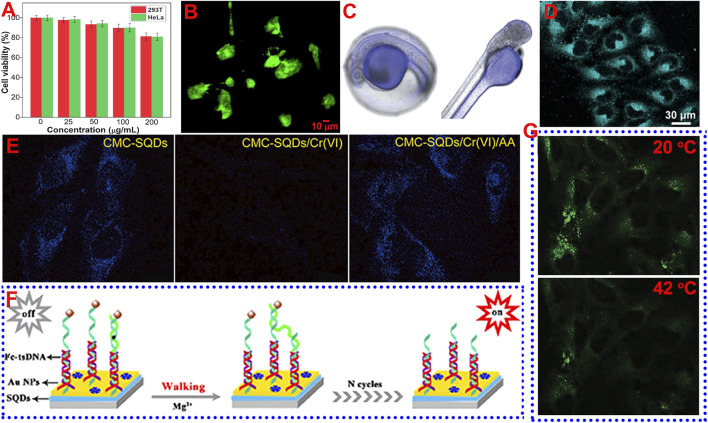
Schematic diagram of the application of SQDs in bioimaging and biosensing. **(A)** Reproduced, with permission, from Duan et al. (2020). Copyright 2020, Elsevier. **(B)** Reproduced, with permission, from Arshad et al. (2021). Copyright 2021, American Chemical Society. **(C)** Reproduced, with permission, from Xia et al. (2021). Copyright 2021, Royal Society of Chemistry. **(D)** Reproduced, with permission, from Gao et al. (2022). Copyright 2022, American Chemical Society. **(E)** Reproduced, with permission, from Duan et al. (2020). Copyright 2020, Elsevier. **(F)** Reproduced, with permission, from Liu et al. (2020). Copyright 2020, American Chemical Society. **(G)** Reproduced, with permission, from Lei et al. (2021). Copyright 2021, Wiley.

Low cytotoxicity and excellent biocompatibility are the prerequisites for the application of fluorophores in bioimaging. As a novel class of metal-free fluorescent QDs, the cytotoxicity evaluation and favorable biocompatible results of SQDs with standard thiazolyl blue tetrazolium bromide (MTT) or cell counting Kit-8 (CCK-8) assay confirmed that they have low cytotoxicity and are suitable for bioapplications. For instance, Zhou’s groups examine the toxicity of different SQDs using MTT assay based on 293T normal cells and HeLa cancer cells. They found that SQDs stabilized with different capping agents (e.g., PEG, CMC, PVA, and HP-β-CD) displayed high cell viability (90%) both for 293T and HeLa cells when the concentration of SQDs was relatively low (0–50 μg/ml) (Duan et al., 2020; Huang et al., 2021a; Lei et al., 2021; Song et al., 2020). When the concentration of SQDs was up to 200 μg/ml, the cell viability still exceeded 80%, suggesting the low cytotoxicity of SQDs (Figure 2A). Wang’s group tested the cytotoxicity of SQDs using CCK-8 assay with BEAS-2B cells (Zhang et al., 2019). After incubation for 24 h, the cell viability was only slightly decreased. When the concentration of SQDs was up to 375 μg/ml, the cell viability was still higher than 85%, suggesting the low cytotoxicity of SQDs. In addition to cytotoxicity evaluation, Zhou et al. also assessed the biocompatibility of SQDs by cultivating soybean seeds in SQDs solution (Gao et al., 2022). The results showed that even in the high concentration of SQDs solution (200 μg/ml), the presence of SQDs had a negligible effect on plant growth, revealing the fine biocompatibility of SQDs. The merits of low toxicity, unique surface characteristics, strong fluorescence, fine colloidal stability, and small size make the SQDs promising for applications in bioimaging and biosensing.

Wang’s group investigated the utilization of SQDs as fluorescent nanoprobe to image living HeLa cells (Qiao et al., 2020). After incubation with SQDs under physiological conditions, the cells were observed using a confocal laser scanning microscope (CLSM), with an excitation wavelength of 405 nm. The cancer cells displayed strong blue fluorescence, suggesting that the SQDs had been internalized into the cells. Wang and co-workers reported that SQDs synthesized by using the ultrasonication method could be used to label BEAS-2B cells (Zhang et al., 2019), and the cytoplasm after labeling could emit strong green fluorescence. Zhou and co-workers incubated SQDs with MCF-7 cancer cells for 2 h and then observed the cells using CLSM. They found that distinct green and yellow fluorescence could be viewed in the cytoplasm of MCF-7 cells under excitation of 458 and 514 nm, respectively (Song et al., 2020). Sk’s group employed green-emissive SQDs with a QY of 4.8% to incubate prostate cancer (Du145) cells (Arshad et al. 2021) (Figure 2B). They found that the SQDs were highly permeable to the Du145 cells. Green fluorescence was visible to both the cytoplasm and nucleus of Du145 cells after incubation with SQDs for 24 h. In addition to cell imaging, SQDs can also be used to label biological tissues. For example, Wang’s group reported the use of SQDs to label zebra fish and larvae (Xia et al., 2021). After incubation with SQDs for 2 h, obvious blue fluorescence could be observed in the yolk of zebra fish embryos (Figure 2C), and the yolk and head of larvae, suggesting that SQDs hold potential for *in vivo* imaging application.

Although SQDs have shown potential in bioimaging, most of the reported SQDs are blue- and green-emissive SQDs. The fluorescence of SQDs may be interfered by the self-fluorescence of cells and biological tissues. In order to overcome this limitation, it is necessary to prepare SQDs with long-wavelength emission. On the other hand, it is also possible to overcome this disadvantage by using the two-photon or multi-photon fluorescence imaging technology. Very recently, Zhou and co-workers used SQDs as a fluorescent probe for two-photon fluorescence imaging of HeLa cells (Gao et al., 2022) (Figure 2D). The HeLa cells can emit bright two-photon fluorescence in the cytoplasm with excitation at 800 nm. On the contrary, no two-photon fluorescence was observed in the cells without incubation with SQDs. However, the utilization of SQDs for two-photon fluorescence imaging is still in its infancy, and more in-depth research is required, such as *in vivo* two-photon fluorescence imaging.

## Biosensing

The development of nanoscience offers great opportunities for biosensing. In this regard, fluorescent SQDs are expected to be attractive fluorescent nanoprobe for biosensing applications because of their excellent fluorescence performance, unique composition, and low toxicity. Until now, the utilization of SQDs as a fluorescent probe for sensing bio-related molecules and metal ions has been intensively studied (Arshad and Palashuddin, 2020; Huang et al., 2021a; Ma et al., 2021; Sheng et al., 2021; Lu et al., 2022; Yan et al., 2022).

Butyrylcholinesterase (BChE) activity is an important indicator of many diseases. Wang and co-workers constructed a sensitive system based on SQDs and MnO_2_ nanosheets to detect BChE (Li et al., 2020). Because the emission spectrum of SQDs overlaps greatly with the absorption spectrum of the MnO_2_ nanosheets, the MnO_2_ nanosheets can effectively quench the fluorescence of SQDs by the inner filter effect (IFE). The addition of BChE can catalyze the formation of acetylcholine into thiocholine, and thereby effectively decompose MnO_2_ nanosheets into Mn^2+^, resulting in the elimination of IFE and the recovery of fluorescence of SQDs. A good linear relationship between the BChE activity and the fluorescence intensity of SQDs in the range of 0.05–10 U/L and 10–500 U/L was observed, and a limit of detection (LOD) of 0.035 U/L was achieved. Zhu’s group also prepared SQDs@MnO_2_ nanocomposites, which can serve as an off–on electrochemiluminescence (ECL) sensor, for the detection of glutathione (GSH) based on the resonance energy transfer (RET) between SQDs and MnO_2_ (Han et al., 2021). When GSH was added, the GSH could reduce Mn^4+^ to Mn^2+^, thus inhibiting the RET effect and recovering the ECL again. As such, SQDs@MnO_2_ could be utilized as a probe to sensitively detect GSH with a low LOD (35 nM) in the concentration range of 0.05–5 μM.

For sensing metal ions, SQDs synthesized under different reaction conditions have presented sensitive and selective fluorescence detection properties for a variety of metal ions, such as Cr(VI) (Duan et al., 2020; Zhang et al., 2020), Fe^3+^ (Huang et al., 2021a; Lei et al., 2021), Co^2+^ (Wang S. et al., 2019), Hg^2+^ (Qiao et al., 2020), and Zn^2+^ ions (Zhao and Fan, 2020). For example, Zhou and co-workers found that the CMC-capped SQDs showed sensitive fluorescence quenching behavior toward Cr(VI) due to the existence of strong IFE. In addition, the quenched fluorescence could be efficiently recovered by ascorbic acid (AA) because the AA can eliminate the IFE by reducing the Cr(VI) to low-valent Cr species. Thus, it is able to employ the CMC-capped SQDs as a fluorescence on–off–on switch to detect Cr(VI) and AA. The detection results showed that the LOD toward Cr(VI) and AA reach 0.024 and 0.18 μM, with a linear range of 0.5–225 μM and 1–300 μM, respectively. In addition to aqueous media, it was also found that SQDs can be utilized to detect Cr(VI) and AA in HeLa cells based on the fluorescence on–off–on phenomenon (Figure 2E). Wang’s group also used SQDs to detect Cr(VI) and AA in zebra fish embryos and larvae, and similar fluorescence on–off–on behavior was observed (Xia et al., 2021).

MicroRNAs are a kind of microcosmic and abundant noncoding RNAs, and there is an urgent need to ultrasensitively detect microRNAs because their abnormal expression was related to many diseases (Chen et al., 2020; Peng L. et al., 2019; Peng X. et al., 2019). Wang and co-worker first reported the employment of SQDs as an ultrasensitive ECL biosensor to detect microRNA-21 (miRNA-21) (Liu et al., 2020). The ECL biosensor was based on an efficient DNA walking machine with triple-stranded DNA (tsDNA) nanostructures as a signal amplifier and SQDs with superior ECL performance as an emitter (Figure 2F). The ECL of SQDs was quenched when ferrocene (Fc)-modified tsDNA was introduced to provide DNA walking orbits. On the other hand, the Fc-modified tsDNA fragments were released under the cleavage of Mg^2+^, resulting in the elimination of the quenching effect and the regeneration of ECL. This ECL biosensor showed excellent sensing performance toward miRNA-21 in the concentration range of 20 aM to 1 nM with a low LOD of 6.67 aM In addition to the detection of molecules and ions, it is also very important to achieve sensitive detection of temperature (Lu and Zhou, 2019; Wang et al., 2016). This is because temperature is a very crucial parameter in a biological environment, which can affect the metabolic process of the organism. Zhou and co-workers prepared an SQDs-based sensor for monitoring temperature in the HeLa cells (Lei et al., 2021). They found that the fluorescence intensity of SQDs decreased with increasing the temperature. For example, after incubation with SQDs, the green fluorescence in the cytoplasm of HeLa cells at 20°C was much brighter than that at 42°C (Figure 2G). This phenomenon that the fluorescence intensity of QDs decreases with the increase in temperature is due to the increase in non-radiative relaxation caused by thermal activation of non-radiative trapping, which is very common in QDs materials (Yu et al., 2012).

So far, the application of SQDs in biosensing has made remarkable progress. However, most SQDs exhibited blue or green fluorescence, which was easily interfered by the autofluorescence of biological samples during detection. On the other hand, these SQDs need to use ultraviolet light as excitation light, which inflicts certain damage to cells and biological tissues. In order to overcome these obstacles, it is necessary to develop SQDs sensors with long-wavelength fluorescence or upconversion fluorescence characteristics.

## Conclusions and Perspectives

In summary, as a new kind of metal-free QDs, fluorescent SQDs have presented great potential in bioimaging and biosensing because of their facile synthetic process, fine aqueous dispersibility and stability, and low toxicity. However, the SQDs also suffer the drawbacks of poor photostability and short emission wavelength, which greatly restrict their real applications. Considering that the imaging process may be interfered by the autofluorescence of biological samples or the samples need to be monitored for a long time, it is of great significance to prepare SQDs with both long-wavelength fluorescence and high photostability. In addition, tailoring the surface of SQDs with functional molecules or doping other atoms to endow the SQDs with more functions, such as targeting function, multiple emission, and environmental responsiveness, is also important but has rarely been reported. Therefore, more efforts are needed to improve the overall performance of SQDs, so as to further promote the application of SQDs in biosensing and bioimaging.
